# Ontogeny and morphological variability of shell in populations of *Leptinaria unilamellata* (d’Orbigny, 1835) (Mollusca, Pulmonata, Subulinidae)

**DOI:** 10.1186/s40064-015-0959-x

**Published:** 2015-04-19

**Authors:** Camilla Medeiros, Roberta Lima Caldeira, Cristiane Lafetá Furtado Mendonça, Omar dos Santos Carvalho, Sthefane D’ávila

**Affiliations:** Laboratório de Helmintologia e Malacologia Médica, Centro de Pesquisas René Rachou, Fiocruz, Av. Augusto de Lima, 1715, Belo Horizonte, MG 30190-002 Brasil; Museu de Malacologia Prof. Maury Pinto de Oliveira, Programa de Pós graduação em Comportamento e Biologia Animal, Departamento de Zoologia, Instituto de Ciências Biológicas, Universidade Federal de Juiz de Fora, Juiz de Fora, MG Brasil

**Keywords:** Allometry, Shell morphometry, Shell shape, Subulinids

## Abstract

**Background:**

Recent studies concerning species of land snails have revealed that the shell morphometrics can provide evidence of the differentiation among populations. In many cases, the morphologic analysis combined with the investigation of molecular variability, can support changes in taxonomy of studied groups. In this sense, the study of shell morphometry during snail development can contribute to the understanding of the structural mechanisms that creates the diversity observed.

**Description:**

The morphological and ontogenetic pattern differences were collected among snails from four different populations, kept under the same laboratorial conditions. It was possible to distinguish characteristic shell morphometrics for snails from each population. The snails from Barra Mansa and Floriano, locations with smaller precipitation indexes presented smaller shell aperture values. The results are discussed in terms of the role of the reproductive strategy of this species as a factor determining shell shape.

**Conclusions:**

Differences in growth allometry indicated that the whole shell forming process is different among the populations, not only the final form of the adult’s shell. Some allometry relationships indicated that, during the snails’ development, the increase in shell width is not proportional to the increase of the width and height of the shell aperture. Thus, there is possibly an antagonism between the adoption of K-strategy and protection against desiccation. Since the spire indices of *L. unilamellata* morphotypes cannot be explained by physical functional aspects, the most likely explanation is the reproductive strategy of this species.

## Introduction

The phylum Mollusca shows great diversity, with more than 110,000 recognized species, most of them possessing a distinct external shell, widely studied by naturalists in the XVII and XVIII centuries (Wilke et al. 2002). The majority of the land snail species have been described simply on the basis of shell characteristics. During the XVIII century, naturalists had already observed variations in shell morphological patterns (Fiorentino et al. 2008). However, it was only from observation that shell shape presents a bimodal distribution that functional aspects of shell morphology began to be investigated (Fiorentino et al. 2008). These functional aspects are related to biotic and abiotic aspects like predation and desiccation risks and shell balance (Dewitt et al. 1999; Chiba and Davison 2007; Okajima and Chiba 2009; Wada and Chiba 2013). Some authors mentioned the importance of the species’ reproductive strategy as a functional aspect relate to shell shape, however the relationship between variation in shell morphology and life history traits was never investigated.

The shell growth takes place during the snail’s development through the addition of material from the mantle collar at the shell’s opening. During this process, the mantle collar movements and also its state of turgidity, determines the shell’s shape and ornamentation variations. Therefore, the variation in the shell formation process, in terms of systematic and regular changes, results in polymorphisms observed in adult individuals (Raup 1961; Patel 2009). In this sense, the study of shell ontogeny allow us to describe the sequence of changes in shell shape, observed during the snail’s development and, as a result, it is possible to understand the structural mechanisms that create the diversity of shapes in different species, as well as the polymorphisms found in one species.

*Leptinaria unilamellata* (d’Orbigny, 1835) is a small terrestrial stylommatophoran, measuring approximately 11 mm long and 5 mm wide, which presents a dextral shell. This species is native to tropical South America (Simone 2006). In Brazil, populations of *L. unilamellata* occur in Amazonas, Pará, Rondônia, Pernambuco, Bahia, Mato Grosso, Minas Gerais, Rio de Janeiro and São Paulo (Araújo 1982). This species can be easily raised in the laboratory and has been used as a model organism in studies concerning biology and morphology (Araújo 1982; Dutra 1988; Almeida and Bessa 2001; Brandolini and Gomes 2002; Carvalho et al. 2009).

The life history of *L. unilamellata* is characterized by great longevity, short juvenile phase, early maturity, repeated reproductive events involving ovoviviparity, small clutch size but large juvenile size relative to parental size (Carvalho et al. 2009). The adoption of a reproductive strategy that features liberation of large juveniles is evidently possible because of the inflated body whorl where the oviduct is located. This species has indeterminate growth, continuing to grow the shell after reproductive maturity. This growth pattern permits a long term investment in reproductive effort since the increase in shell size (specially the body whorl) permits enlargement of the uterus and as a consequence, accommodates more juveniles.

Tryon and Pilsbry (1906) considered the species diagnosis of the genus *Leptinaria* difficult, since their shells present considerable variation. These authors highlighted, that the determination of *Leptinaria* species could be only made upon the achievement of studies with a good series of young and adult specimens. Most of the original descriptions of *Leptinaria* species, including *L. unilamellata*, were limited to shell morphology. In this sense, it is possible that the observed differences in the shell, on which justified the creation of a new species, may represent in reality, variation or indeed polymorphisms in a single species, or different developmental phases. On the other hand, the existence of complexes of cryptic species might have been neglected due to the superficial similarities among specimens identified only on the basis of shell morphology.

Recent studies concerning some species of land snails have revealed that the shell morphometrics can provide evidence of differentiation among populations (Conde- Padín et al. 2009; Fiorentino et al. 2008). In many cases, morphologic analysis combined with molecular and/or anatomical data, can support changes in taxonomy of the studied groups (Raahauge and Kristensen 2000; Choh et al. 2006), for example, descriptions of new species from the detection of species complexes, or in other hand, the reduction of recognized species number by synonymy, based on the finding that the variation observed corresponds to a polymorphism (Paraense 1976; Carvajal-Rodriguez et al. 2005; Velasquez 2006; Carstensen et al. 2009).

The aim of this work was to describe the shell ontogeny of *L. unilamellata* and determine if snails from different populations differ in shell morphology and also how variations are relate to shell formation process.

## Methodology

### Snails

**Figure 1:**
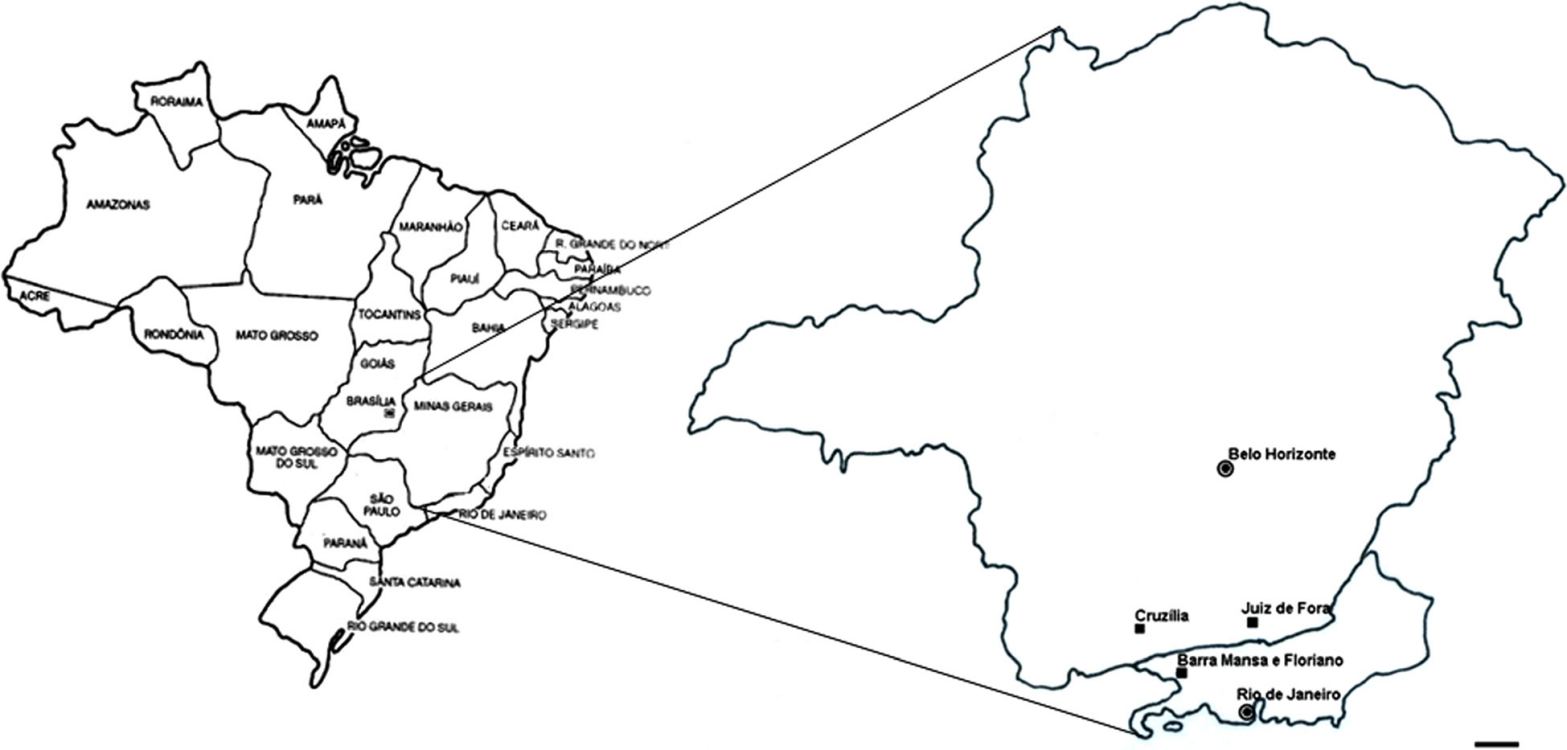
Geographical location of the municipalities of *Leptinaria unilamellata* collected. Scale bar: 100 km.

Laboratory colonies were established from specimens of *L. unilamellata* collected at the following locations: municipality of Juiz de Fora, Minas Gerais (MG) (21°44′29.65″S, 43°21′19.65″W); municipality of Cruzília, MG (21°50′20″S, 44°48′28″W); municipality of Barra Mansa, Rio de Janeiro (RJ) (22°31′59.14″S 44°9′54.21″W) and Floriano, district of Barra Mansa, RJ (22°31′59.14″S 44°9′54.20″W) (Figure 1). Snails from each location were fixed, dissected and morphologically identified as *L. unilamellata* as described by Araújo (1982), Carvalho et al. (2009) and Medeiros et al. (2013). Each laboratory colony comprised snails from a single same location; they were kept at room temperature and under natural lighting in transparent plastic boxes (14 cm in diameter, 9 cm in height). The bottom of each box was lined with humus, which was moistened every 2 days with 10 ml of tap water. The snails were fed *ad libitum* with a commercial poultry feed, supplemented with a premix of minerals, vitamins and calcium carbonate (Bessa and Araújo 1995a,b). When the laboratory colony included at least five generations from the original field collection, newly hatched snails were randomly taken and placed in new transparent plastic boxes under the same conditions as described above, with 30 individuals per box. These individuals were used in the morphologic study.

### Shell description

We described the general shell morphology of *L. unilamellata* specimens from each location at the age of 180 days. The shells were drawn under an Olympus stereoscopic microscope with camera lucida and deposited at the Museum of Malacology Prof. Maury Pinto de Oliveira, of the Federal University of Juiz de Fora, Juiz de Fora, Brazil. Shells’ pictures were acquired using a Sony digital camera (Cybershot 8.1 DSC-H3).

### Shell morphometric

**Figure 2:**
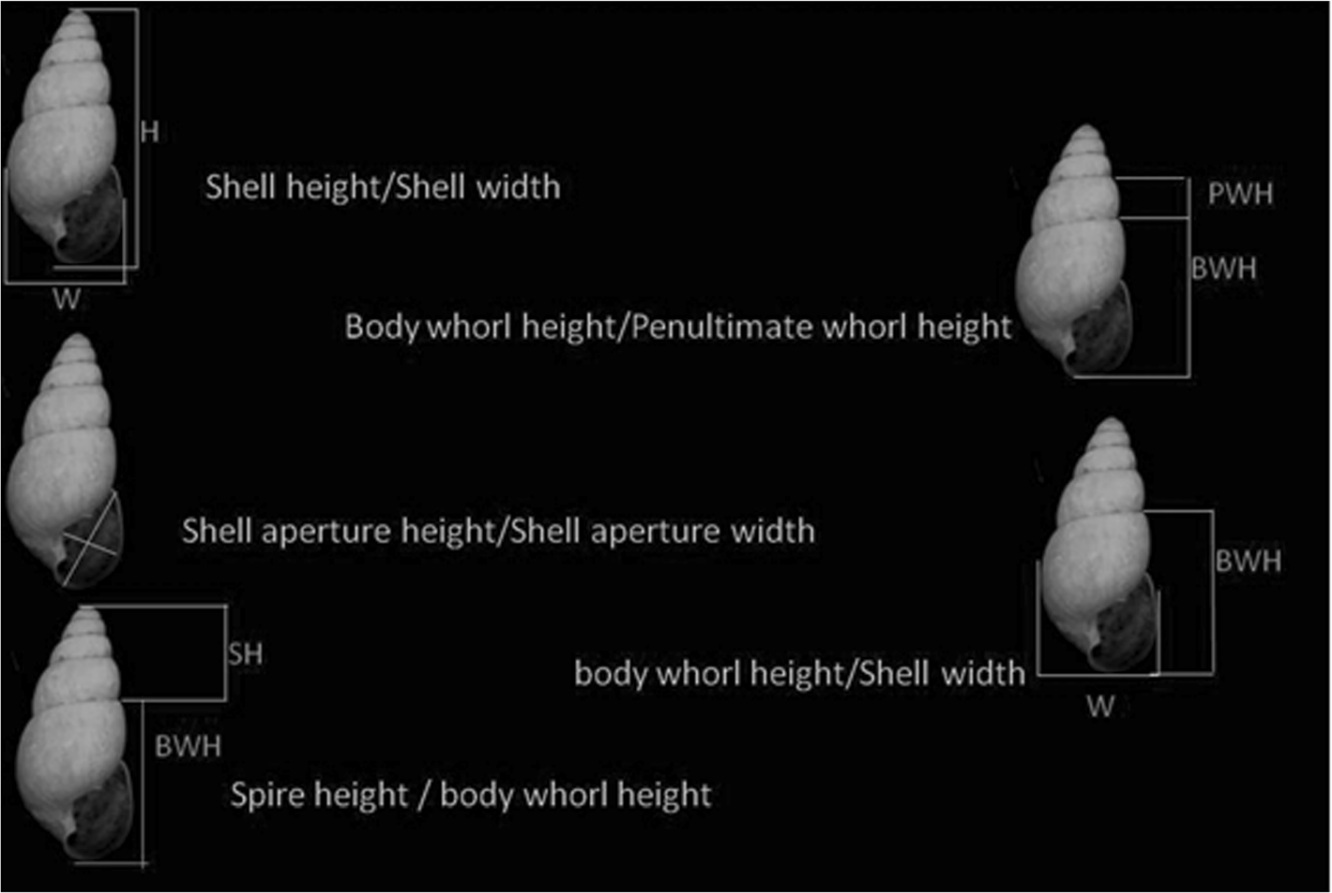
Linear measurements taken from populations of *Leptinaria unilamellata*.

Shells from 30 snails of each locality, with ages of 30, 60, 90, 120, 150 and 180 days were measured with a Hardened Stainless 1/28 in 1/20 mm caliper 0.01 mm precision. The following linear measurements were taken: shell height (H), shell width (W), shell aperture height (AH), shell aperture width (AW), body whorl height (BH), penultimate whorl height (PH) and spire height (SH) (Figure 2). The number of shell’s whorls (NW) was also counted. These measurements followed the methodology proposed by Chiu et al. (2002). From the values obtained for each linear measurement, the following ratios were calculated: shell height/shell width (H/W); body whorl height/penultimate whorl height (BH/PH); body whorl height/shell width (BH/W); shell aperture height/shell aperture width (AH/AW) and spire height/ body whorl height (SH/BH). All of the statistical analyses were applied for each age observed. We performed all the statistical tests through the program Bioestat 5.0. Prior to analyses, the values of shell linear measurements and ratios were subjected to logarithmic transformation (log_10_), to minimize the normality deviations.

### Interpopulational variation analysis

In order to detect variations in shell morphology between specimens from each location at different ages, the mean values of linear measurements and ratios were compared through the variance analysis (ANOVA, p ≤ 0.05), followed by t-test. In order to identify similarities of the shell morphometry between snails from each location, values of Euclidean distance were calculated and cluster analysis performed by the Ward aggregation method (Hórsak et al. 2007 modified), taking into account the minimum variance among the groups. In order to distinguish the groups from the different locations based on the measured variables, a discriminant function analysis was performed (Conde- Padín et al. 2007 modified).

### Growth allometry analysis

The study of shell growth allometry was performed with the snails from each location at different ages, through linear regression analysis of the general equation y = a . x^b^ (“a” is the intercept and “b” the allometric coefficient) for the following relationships: 1: AW = a . W^b^ (Shell aperture width = a . Shell width^b^); 2: AH = a . W^b^ (Shell aperture height = a . Shell width^b^); 3: SH = a . W^b^ (Spire height = a . Shell width^b^); 4: H = a . W^b^ (Shell height = a . Shell width^b^); 5: SH = a . H^b^ (Spire height = a . Shell height^b^) and 6: AH = a . H^b^ (Shell aperture height = a . Shell height^b^) (Chiu et al. 2002 modified). The allometric coefficient was used with the objective to supply the proportion of the “y” variability which can be explained by “x”. The shell growth was considered positively allometric when b > 1, negatively allometric when b <1 or isometric when b = 1. To determine the statistical significance of values of the allometric coefficient (b) the t-test was performed, with significance level of p < 0.05. We considered only the significant allometric values of coefficient (b) as showed by the t-test.

## Results

The Table 1 shows the main qualitative characteristics for the morphotypes differentiation. These characteristics are not mutually exclusive and therefore not sufficient to separate these morphotypes in distinct species.

**Table 1:** **Main shell characteristics of different populations of** ***Leptinaria unilamellata***

**Location**	**Columella**	**Shell aperture**	**Lamella**	**Body whorl**	**Whorls height**	**Spire**	**Sutures**
**Juiz de Fora**	Square	Round	Presented or not, low	Wide and round	Decreases gradually	Prominent or low	First two sutures are deep
**Cruzília**	Triangular	Wide and round, dislocated	Prominent and well developed	Wide and round	Penultimate whorl possesses a larger height	Low	Quite deep
**Floriano**	Triangular	Wide and oval	Shallow	Round and uniform	Penultimate and last whorls have larger height	Prominent	Smooth (except for the first)
**Barra Mansa**	Triangular	Narrow and oval	Smooth	Round, little globular	Decreases gradually	Prominent	Smooth

### Interpopulational variation

The relationships among the variables and differences between the values of linear measurements and calculated measurement ratios from different morphotypes varied in the course of snails’ development, due to differences in the growth patterns (Tables 2 and 3). Thus, for distinction among the four morphotypes, we considered only the values observed at the age of 180 days, when the snail’s growth was established. The ANOVA demonstrated that all variables differed statistically among the snails from the different locations at specific ages. The average values of shell height, shell aperture height and body whorl height were significantly different in snails from all locations (Table 4).

**Table 2:** **Average values, standard error and amplitude (minimum and maximum) of linear measurements (mm) taken from individuals of** ***Leptinaria unilamellata*** **from Juiz de Fora, Cruzília, Barra Mansa and Floriano locations**

	**30 days**	**60 days**	**90 days**	**120 days**	**150 days**	**180 days**
***Leptinaria unilamellata*** **from the Juiz de Fora location**						
H	4.4 ± 0.4 (3.8 – 5.1)	6.7 ± 0.5 (5.8 - 7.8)	12.4 ± 1.0 (10.2 - 14.8)	13.4 ± 1.5 (11.3 - 16)	13.5 ± 1.6 (10.8 – 16.7)	13.3 ± 1.3 (11.6 - 15.8)
W	2.8 ± 0.2 (2.3 – 3.4)	3.8 ± 0.2 (3.4 - 4.2)	5.8 ± 0.3 (5.2 - 6.4)	6.1 ± 0.6 (5–7.2)	6.2 ± 0.7 (5–7.6)	6.3 ± 0.6 (5–7.4)
AH	2.1 ± 0.3 (1.5 - 2.7)	3.3 ± 0.3 (2.7 - 3.9)	4.4 ± 0.3 (3.8 - 5)	5.3 ± 0.6 (4.5 - 7.1)	5.3 ± 0.7 (4–6.6)	5.2 ± 0.5 (4–6)
AW	1.3 ± 0.2 (0.9 - 1.6)	1.8 ± 0.2 (1.4 - 2.2)	2.7 ± 0.2 (2.2 - 3.1)	2.9 ± 0.3 (2.2 - 3.5)	3.2 ± 0.4 (2.4 - 3.9)	3.4 ± 0.6 (2.2 - 4.4)
BH	3.5 ± 0.3 (2.9 - 4)	5.1 ± 0.3 (4.6 - 5.9)	7.7 ± 0.4 (6.7 - 8.6)	7.9 ± 0.7 (6.4 - 9.1)	7.9 ± 0.7 (7–9.3)	8 ± 0.6 (6.8 - 9.3)
PH	1.0 ± 0.1 (0.7 - 1.1)	1.4 ± 0.2 (1.2 - 1.9)	2.6 ± 0.2 (1.9 - 3.2)	2.8 ± 0.3 (2.3 - 3.3)	3.1 ± 0.4 (2.5 - 4)	2.9 ± 0.3 (2.4 - 3.4)
SH	1.2 ± 0.2 (0.8 - 1.6)	2.0 ± 0.2 (1.6 - 2.6)	5.0 ± 0.5 (4.1 - 6.1)	5.5 ± 0.7 (4.4 - 6.8)	5.7 ± 0.8 (4.4 - 7.1)	5.7 ± 0.8 (4.3 - 7.3)
NW	3.34 ± 0.22 (2.75 - 3.75)	3.98 ± 0.34 (3.5 - 5)	5.14 ± 0.31 (4.5 - 5.75)	5.5 ± 0.35 (5–6.5)	5.81 ± 0.47 (5–6.5)	5.68 ± 0.45 (5–6.75)
***Leptinaria unilamellata*** **from the Cruzília location**						
H	3.3 ± 0.3 (2.8 - 4.4)	9.7 ± 1.2 (6.8 - 12.2)	13.2 ± 1.8 (8.9 - 15.7)	11.8 ± 0.7 (10.5 – 13.4)	15.4 ± 1.5 (12.9 - 18)	14.9 ± 0.8 (13–16.6)
W	2.2 ± 0.2 (1.8 - 2.7)	5 ± 0.5 (4–6)	6.3 ± 0.7 (4.7 - 7.2)	5.6 ± 0.4 (4.7 - 6.5)	7.3 ± 0.6 (6.1 - 8.4)	6.7 ± 0.6 (5.3 - 7.8)
AH	1.8 ± 0.3 (1.2 - 2.5)	4.4 ± 0.5 (3.2 - 5.1)	5.4 ± 0.7 (3.6 - 6.8)	4.8 ± 0.3 (4.3 - 5.8)	5.8 ± 0.6 (4.7 - 7)	5.8 ± 0.7 (4–7.4)
AW	1.1 ± 0.2 (0.6 - 1.4)	2.2 ± 0.3 (1.4 - 2.8)	2.8 ± 0.4 (2.1 - 3.8)	2.5 ± 0.3 (1.6 - 3.3)	3.6 ± 0.4 (2.8 - 4.4)	4.2 ± 0.5 (3–5.1)
BH	2.7 ± 0.3 (2.3 - 3.9)	6.9 ± 0.6 (5.5 - 8)	8.6 ± 0.1 (6.3 - 9.9)	7.8 ± 0.4 (7–8.7)	9.5 ± 0.1 (5.7 - 11)	8.7 ± 1.4 (4.3 – 10.4)
PH	0.7 ± 0.1 (0.6 - 0.8)	2.5 ± 0.3 (2–3.4)	3.2 ± 0.5 (2.1 - 3.9)	2.8 ± 0.2 (2.4 - 3.2)	3.6 ± 0.5 (2.9 - 4.5)	3.7 ± 1.1 (2.9 - 8.8)
SH	0.8 ± 0.1 (0.5 - 1)	3.3 ± 0.5 (2.5 - 4.6)	4.8 ± 0.8 (3.1 - 5.9)	4.1 ± 0.3 (3.6 - 4.7)	6 ± 1.3 (4.4 - 11.3)	5.7 ± 0.4 (5–6.5)
NW	3.06 ± 0.12 (3–3.5)	4.25 ± 0.38 (3.5 - 5.25)	5.08 ± 0.43 (4–5.75)	4.93 ± 0.22 (4.5 - 5.25)	5.68 ± 0.3 (5.25 - 6.25)	5.5 ± 0.29 (5.5 - 6)
***Leptinaria unilamellata*** **from the Barra Mansa location**						
H	4.1 ± 0.2 (3.6 - 4.6)	10.4 ± 0.5 (9.1 - 11.9)	11.2 ± 0.4 (10.5 - 12.8)	12.1 ± 0.7 (10.8 - 13.4)	12.5 ± 0.4 (11.7 - 13.3)	11.9 ± 0.5 (11–13.1)
W	2.5 ± 0.1 (2.3 - 2.7)	4.8 ± 0.2 (4.4 - 5.3)	4.9 ± 0.2 (4.6 - 5.3)	5.1 ± 0.2 (4.5 - 5.4)	5.4 ± 0.2 (5–6)	5.2 ± 0.2 (4.5 - 5.6)
AH	1.8 ± 0.2 (2.3 - 1.4)	4.1 ± 0.3 (3.4 - 5.1)	3.9 ± 0.3 (3.4 - 4.7)	4.1 ± 0.3 (3.6 - 5.5)	4.4 ± 0.4 (3.8 - 5.1)	4.4 ± 0.3 (3.7 - 5.3)
AW	1.2 ± 0.2 (0.9 - 1.4)	2.5 ± 0.5 (1.6 - 3.8)	2.5 ± 0.1 (2.2 - 2.9)	2.6 ± 0.2 (2.2 - 2.9)	3.0 ± 0.3 (2.4 - 3.7)	2.9 ± 0.4 (2.1 - 4)
BH	3.1 ± 0.2 (2.7 - 3.5)	6.8 ± 0.4 (6–7.4)	7.2 ± 0.2 (6.8 - 7.8)	7.4 ± 0.3 (6.7 - 7.9)	7.3 ± 0.4 (5.5 - 7.8)	7.3 ± 0.2 (6.6 - 7.6)
PH	0.9 ± 0.1 (0.7 - 1.1)	2.7 ± 0.3 (2–3.3)	2.5 ± 0.2 (2.1 - 3)	2.7 ± 0.2 (2.3 - 3.1)	2.8 ± 0.2 (2.4 - 3)	2.8 ± 0.3 (2.1 - 3.3)
SH	1.2 ± 0.1 (1–1.4)	4.1 ± 0.3 (3.6 - 5.2)	4.3 ± 0.3 (3.8 - 5.4)	4.7 ± 0.4 (4–5.7)	5.1 ± 0.3 (4.7 - 5.8)	4.8 ± 0.5 (4–6)
NW	3.45 ± 0.22 (2.75 - 3.75)	4.9 ± 0.24 (4.5 - 5.75)	5.78 ± 0.27 (5.25 - 6.25)	5.87 ± 0.27 (5–6.25)	5.44 ± 0.30 (5–6)	5.18 ± 0.30 (4.5 - 6)
***Leptinaria unilamellata*** **from the Floriano location**						
H	4 ± 0.3 (3.5 - 4.5)	6.1 ± 0.7 (4.7 - 7.2)	10.5 ± 0.5 (9.5 - 11.6)	10.9 ± 0.3 (10.2 - 11.6)	11.3 ± 0.5 (10.5 - 12.8)	11.4 ± 0.6 (10.2 - 12.3)
W	2.4 ± 0.1 (2.2 - 2.7)	2.5 ± 0.2 (2–2.9)	4.8 ± 0.3 (3.8 - 5.4)	4.8 ± 0.2 (4.4 - 5.3)	5 ± 0.2 (4.4 - 5.5)	5 ± 0.2 (4.6 - 5.4)
AH	1.7 ± 0.2 (1.3 - 2.1)	3.3 ± 0.3 (2.8 - 4.1)	4 ± 0.5 (2.9 - 5)	4.1 ± 0.4 (2.8 - 4.8)	4 ± 0.4 (3–4.7)	4.1 ± 0.2 (3.6 – 4.7)
AW	1.1 ± 0.2 (0.8 - 1.6)	1.5 ± 0.2 (1.1 - 1.8)	2.2 ± 0.3 (1.6 - 3)	2.4 ± 0.3 (2–3)	2.8 ± 0.3 (2.2 - 3.6)	2.7 ± 0.3 (2.3 - 3.4)
BH	3 ± 0.2 (2.7 - 3.3)	4.2 ± 0.4 (3.6 - 5.2)	6.6 ± 0.5 (5.6 - 7.7)	6.5 ± 0.3 (5.4 - 6.9)	6.8 ± 0.3 (5.9 - 7.6)	6.8 ± 0.3 (6.4 - 7.6)
PH	0.8 ± 0.1 (0.6 - 0.9)	1.4 ± 0.2 (0.9 - 1.8)	2.9 ± 0.3 (2.4 - 3.8)	2.8 ± 0.2 (2.3 - 3.3)	2.7 ± 0.2 (2.2 - 3.1)	2.8 ± 0.2 (2.4 - 3.3)
SH	1.1 ± 0.1 (0.9 - 1.5)	2 ± 0.3 (1.3 - 2.5)	4.1 ± 0.3 (3–4.7)	4.5 ± 0.3 (3.6 - 5.7)	4.6 ± 0.2 (4.1 - 5.1)	4.7 ± 0.3 (4–5.4)
NW	3.4 ± 0.22 (3–4)	4.4 ± 0.38 (3.9 - 5.15)	5.03 ± 0.35 (4.25 - 6.25)	5.14 ± 0.18 (4.75 - 5.5)	5.29 ± 0.19 (5–5.75)	5.41 ± 0.26 (5–6)

Legend: H - shell height; W - shell width; AH - shell aperture height; AW - shell aperture width; BH - body whorl height; PH - penultimate whorl height; SH - spire height; NW - number of shell’s whorls.

**Table 3:** **Average values, standard error and amplitude (minimum and maximum) of ratios between linear measurements (mm) taken from individuals of** ***Leptinaria unilamellata*** **from Juiz de Fora, Cruzília, Barra Mansa and Floriano locations**

	**30 days**	**60 days**	**90 days**	**120 days**	**150 days**	**180 days**
***Leptinaria unilamellata*** **from the Juiz de Fora location**						
H/W	1.58 ± 0.08 (1.41 - 1.75)	1.77 ± 0.07 (1.64 - 1.91)	2.13 ± 0.11 (1.95 - 2.43)	2.18 ± 0.09 (1.98 - 2.38)	2.15 ± 0.12 (1.89 - 2.49)	2.14 ± 0.12 (1.8 - 2.49)
BH/PH	3.74 ± 0.43 (3–4.71)	3.65 ± 0.29 (3.11 - 4.33)	3 ± 0.22 (2.54 - 3.53)	2.84 ± 0.29 (2.24 - 3.6)	2.6 ± 0.25 (2.13 - 3.12)	2.74 ± 0.27 (1.18 - 3.32)
BH/W	1.25 ± 0.06 (1.15 - 1.38)	1.36 ± 0.06 (1.24 - 1.59)	1.3 ± 0.04 (1.23 - 1.41)	1.29 ± 0.04 (1.21 - 1.4)	1.27 ± 0.06 (1.18 - 1.43)	1.27 ± 0.06 (1.18 - 1.45)
AH/AW	1.70 ± 0.22 (1.36 - 2.22)	1.85 ± 0.18 (1.64 - 2.64)	0.77 ± 0.04 (0.67 - 0.86)	1.83 ± 0.23 (1.34 - 2.32)	1.67 ± 0.24 (1.29 - 2.22)	1.57 ± 0.21 (1.19 - 2.27)
SH/BH	0.35 ± 0.04 (0.26 - 0.4)	0.39 ± 0.03 (0.34 - 0.45)	0.66 ± 0.05 (0.54 - 0.77)	0.7 ± 0.05 (0.59 - 0.81)	0.71 ± 0.05 (0.63 - 0.84)	0.71 ± 0.07 (0.56 - 0.91)
***Leptinaria unilamellata*** **from the Cruzília location**						
H/W	1.55 ± 0.09 (1.44 - 1.91)	1.93 ± 0.11 (1.58 - 2.35)	2.09 ± 0.09 (1.83 - 2.22)	2.09 ± 0.11 (1.97 - 2.43)	2.10 ± 0.08 (1.94 - 2.27)	2.16 ± 0.17 (2.05 - 2.75)
BH/PH	3.99 ± 0.57 (3.13 - 5.57)	2.76 ± 0.25 (2.35 - 3.26)	2.75 ± 0.39 (1.8 - 4.05)	2.76 ± 0.2 (2.42 - 3.27)	2.66 ± 0.3 (2.39 - 3.13)	2.51 ± 0.57 (2.75 - 2.97)
BH/W	1.23 ± 0.11 (1.10 - 1.7)	1.36 ± 0.05 (1.22 - 1.54)	1.37 ± 0.07 (1.3 - 1.45)	1.38 ± 0.06 (1.29 - 1.6)	1.29 ± 0.12 (1.21 - 1.4)	1.31 ± 0.24 (1.11 - 1.74)
AH/AW	1.7 ± 0.22 (1.21 - 2.27)	1.99 ± 0.2 (1.70 - 2.59)	1.95 ± 0.33 (1.42 - 2.87)	1.99 ± 0.31 (1.45 - 2.83)	1.61 ± 0.22 (1.23 - 2.14)	1.41 ± 0.22 (0.95 - 2.13)
SH/BH	3 ± 0.04 (0.19 - 0.37)	0.48 ± 0.04 (0.34 - 0.58)	0.55 ± 0.05 (0.46 - 0.68)	0.53 ± 0.03 (0.49 - 0.6)	0.64 ± 0.15 (0.5 - 1.28)	0.69 ± 0.2 (0.55 - 1.4)
***Leptinaria unilamellata*** **from the Barra Mansa location**						
H/W	1.69 ± 0.1 (1.56 - 2)	2.18 ± 0.1 (1.89 - 2.41)	2.28 ± 0.07 (2.19 - 2.51)	2.39 ± 0.09 (2.22 - 2.59)	2.3 ± 0.09 (2.17 - 2.56)	2.3 ± 0.13 (2.07 - 0.28)
BH/PH	3.69 ± 0.3 (3.1 - 4.57)	2.59 ± 0.3 (2.0 - 3.3)	2.88 ± 0.18 (2.43 - 3.29)	2.79 ± 0.13 (2.55 - 3.04)	2.59 ± 0.2 (2.04 - 3.08)	2.66 ± 0.3 (2.12 - 3.62)
BH/W	1.27 ± 0.05 (1.17 - 1.39)	1.43 ± 0.09 (1.25 - 1.68)	1.48 ± 0.05 (1.37 - 1.61)	1.47 ± 0.04 (1.40 - 1.58)	1.35 ± 0.08 (1.02 - 1.5)	1.4 ± 0.07 (1.22 - 1.62)
AH/AW	1.59 ± 0.23 (1.23 - 2.11)	1.7 ± 0.36 (1.08 - 2.68)	1.55 ± 0.14 (1.31 - 2.04)	1.58 ± 0.12 (1.38 - 0.19)	1.52 ± 0.21 (1.15 - 1.92)	1.58 ± 0.3 (1–2.52)
SH/BH	0.39 ± 0.03 (0.33 - 0.45)	0.6 ± 0.06 (0.49 - 0.74)	0.59 ± 0.03 (0.69 - 0.54)	0.64 ± 0.05 (0.53 - 0.72)	0.7 ± 0.07 (0.63 - 1.05)	0.67 ± 0.06 (0.57 - 0.81)
***Leptinaria unilamellata*** **from the Floriano location**						
H/W	1.64 ± 0.11 (1.42 - 2.05)	2.46 ± 0.16 (2.14 - 2.76)	2.19 ± 0.14 (1.9 - 2.58)	2.27 ± 0.08 (2.11 - 2.46)	2.28 ± 0.1 (2.07 - 2.47)	2.28 ± 0.1 (2.12 - 2.51)
BH/PH	3.85 ± 0.4 (3.11 - 4.57)	3.19 ± 0.48 (2.17 - 4.67)	2.33 ± 0.3 (1.66 - 2.96)	2.37 ± 0.24 (1.8 - 2.83)	2.58 ± 0.26 (2.16 - 3.23)	2.48 ± 0.2 (2.03 - 2.92)
BH/W	1.25 ± 0.06 (1.08 - 1.36)	1.72 ± 0.17 (1.39 - 2.05)	1.38 ± 0.13 (1.12 - 1.71)	1.35 ± 0.09 (1.08 - 1.53)	1.38 ± 0.08 (1.18 - 1.56)	1.36 ± 0.07 (1.28 - 1.65)
AH/AW	1.47 ± 0.18 (1.18 - 2)	2.25 ± 0.27 (1.83 - 3.15)	1.84 ± 0.35 (1.21 - 2.73)	1.74 ± 0.27 (1.27 - 2.4)	1.47 ± 0.22 (1.07 - 1.96)	1.54 ± 0.18 (1.16 - 1.88)
SH/BH	0.37 ± 0.04 (0.3 - 0.48)	0.47 ± 0.08 (0.34 - 0.64)	0.63 ± 0.06 (0.49 - 0.79)	0.7 ± 0.06 (0.56 - 0.88)	0.68 ± 0.05 (0.57 - 0.78)	0.69 ± 0.04 (0.61 – 0.78)

Legend: H/W: shell height/shell width; BH/PH: body whorl height/penultimate whorl height; BH/W: body whorl height/shell width; AH/AW: shell aperture height/shell aperture width; SH/BH: spire height/ body whorl height.

**Table 4:** **Variance analysis (ANOVA) of shell morphometry of** ***Leptinaria unilamellata*** **from localities of Juiz de Fora, Cruzília, Barra Mansa e Floriano, with ages of 30, 60, 90, 120, 150 and 180 days**

**ANOVA**												
**Shell morphometry**												
	**30 days**		**60 days**		**90 days**		**120 days**		**150 days**		**180 days**	
	**F**	**P**	**F**	**p**	**F**	**p**	**F**	**p**	**F**	**p**	**F**	**p**
H	68.55	0.0001*	216.9	0.0001*	37.47	0.0001*	39.84	0.0001*	69.64	0.0001*	96.36	0.0001*
W	71.29	0.0001*	395.34	0.0001*	91.69	0.0001*	71.26	0.0001*	142.31	0.0001*	104.87	0.0001*
AH	18.03	0.0001*	68.67	0.0001*	62.82	0.0001*	49.25	0.0001*	67.29	0.0001*	80.30	0.0001*
AW	6.22	0.0009*	63.15	0.0001*	22.16	0.0001*	17.04	0.0001*	28.70	0.0001*	56.14	0.0001*
BH	53.59	0.0001*	231.51	0.0001*	52.52	0.0001*	50.83	0.0001*	79.55	0.0001*	32.67	0.0001*
PH	42.66	0.0001*	217.76	0.0001*	27.05	0.0001*	39.84	0.0001*	42.88	0.0001*	15.82	0.0001*
SH	55.83	0.0001*	221.02	0.0001*	20.05	0.0001*	42.95	0.0001*	17.43	0.0001*	29.22	0.0001*
NW	23.83	0.0001*	37.72	0.0001*	60.27	0.0001*	69.95	0.0001*	13.65	0.0001*	11.78	0.0001*

Legend: H - shell height; W - shell width; AH - shell aperture height; AW - shell aperture width; BH - body whorl height; PH - penultimate whorl height; SH - spire height; NW - number of shell’s whorls. *:significant differences between the averages.

The snails’ shells of Barra Mansa and Floriano locations presented more similar morphometry (Table 2). However, the average values of shell height, shell aperture height and body whorl height were significantly larger for Barra Mansa, while the Floriano shells had a greater average number of whorls. The Barra Mansa shells had greater values for height/shell width, aperture height/shell aperture width, body whorl height/penultimate whorl height and body whorl height/shell width compared with populations of Floriano (Table 3). The snails’ shells of Juiz de Fora were similar to those of Barra Mansa and Floriano only in the penultimate whorl height. However, the Juiz de Fora shell had a greater number of whorls. Moreover, the body whorl height of Juiz de Fora shells were larger compared to other shells (Table 2). The spire height/body whorl height ratio for Juiz de Fora shell was near 1:1.71, while the same ratio was near 1:1.69 for Floriano and Cruzília and 1:1.67 for Barra Mansa shells. The average values of the variables of Cruzília shells were closer to the values observed in Juiz de Fora morphotype. The Cruzília morphotype shells showed a significantly higher body whorl than the morphotypes from other locations. Moreover, Cruzília morphotypes presented a smaller number of whorls compared to Juiz de Fora morphotype (Table 2). However, the Cruzília shells present a significantly greater shell width and smaller values of the ratio shell height/shell width. The shells from all locations exhibited the relationship between height and width of the shell aperture near 1:1.5 (Juiz de fora = 1:1.57; Cruzília = 1:1.41; Barra Mansa = 1:1.58 and Floriano = 1:1.54) (Table 3).

The Euclidean distance analysis showed smaller distances between Barra Mansa and Floriano shells and the majority of higher distances between Floriano and Cruzília in 90 (6.0936), 120 (6.1757) and 180 days (6.954) and between Floriano and Juiz de Fora shells in 150 days (7.2691). The Barra Mansa and Floriano shells were more similar in almost all ages, except for age of 60 days (higher distance of 6.1924), these shells presented smaller values of Euclidean distance, when compared to the shells from other locations. This was confirmed by the progressive decrease of the Euclidean distance between these two locations at the ages of 120 (3.6413), 150 (1.9127) and 180 days (1.5697). The shells from Cruzília and Juiz de Fora showed similarities in almost all observed ages, except for age of 30 days (higher distance of 6.9502). In almost all ages, except for 60 days, these shells were separated by rather small Euclidean distances, when compared to shells from the other two locations. The shells from Cruzília and Juiz de Fora showed similarities in almost all examined ages, except for age of 30 days.

**Figure 3:**
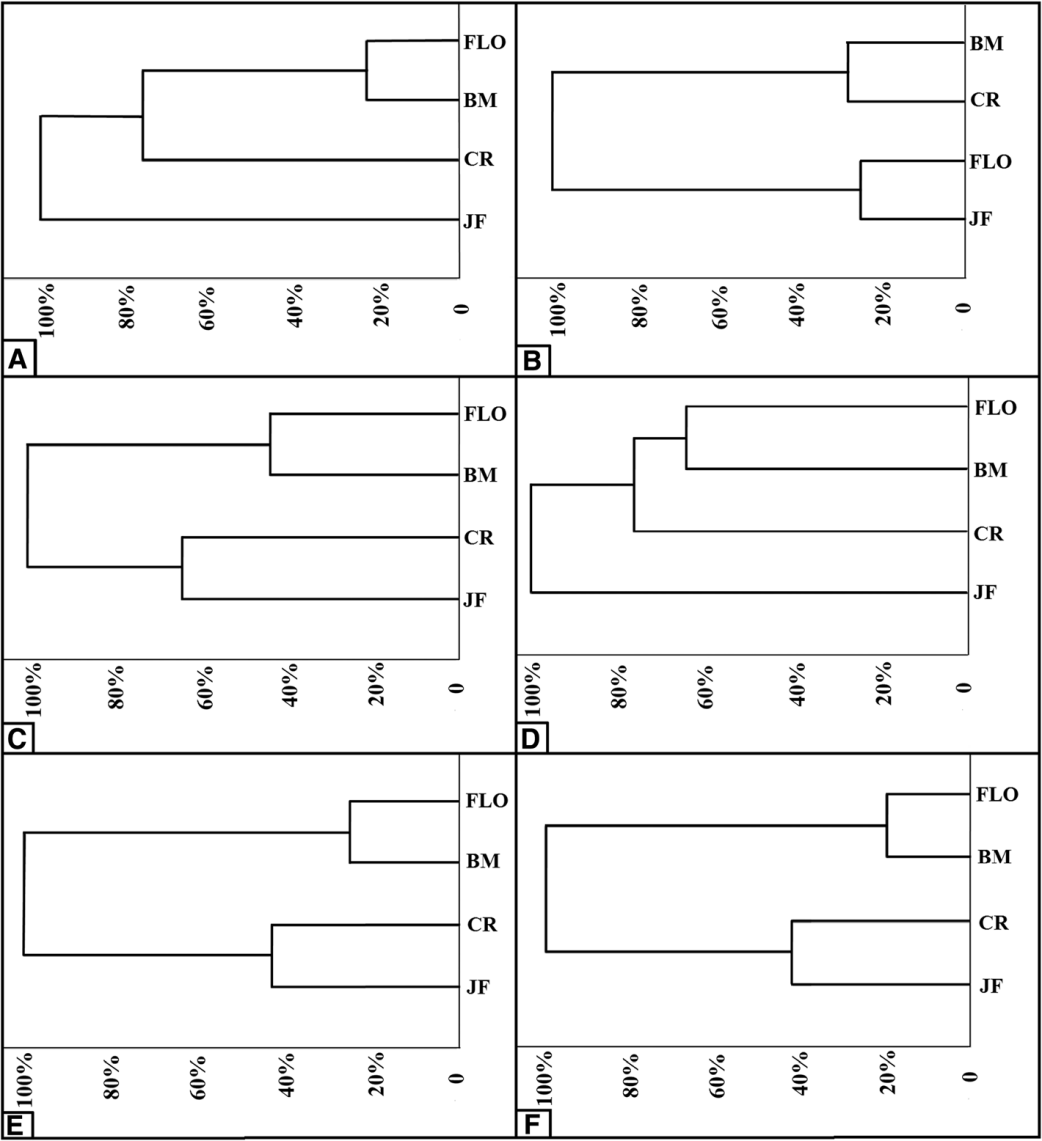
Cluster analysis of shell morphometry of *Leptinaria unilamellata* from localities of Juiz de Fora (JF), Cruzília (CR), Barra Mansa (BM) and Floriano (FLO), with ages of 30 **(A)**, 60 **(B)**, 90 **(C)**, 120 **(D)**, 150 **(E)** and 180 **(F)** days.

The cluster analysis (Figure 3) showed that Barra Mansa and Floriano shells were clustered in the ages of 30 (distance of 23%), 90 (44%), 120 (64%), 150 (25%) and 180 (20%). The Juiz de Fora and Cruzília shells were clustered at the ages of 90, 150 and 180 days, with distances of 64%, 43% and 43% respectively. At these ages, the distance between Juiz de Fora and Cruzília shells were higher than those between Barra Mansa and Floriano shells.

**Figure 4:**
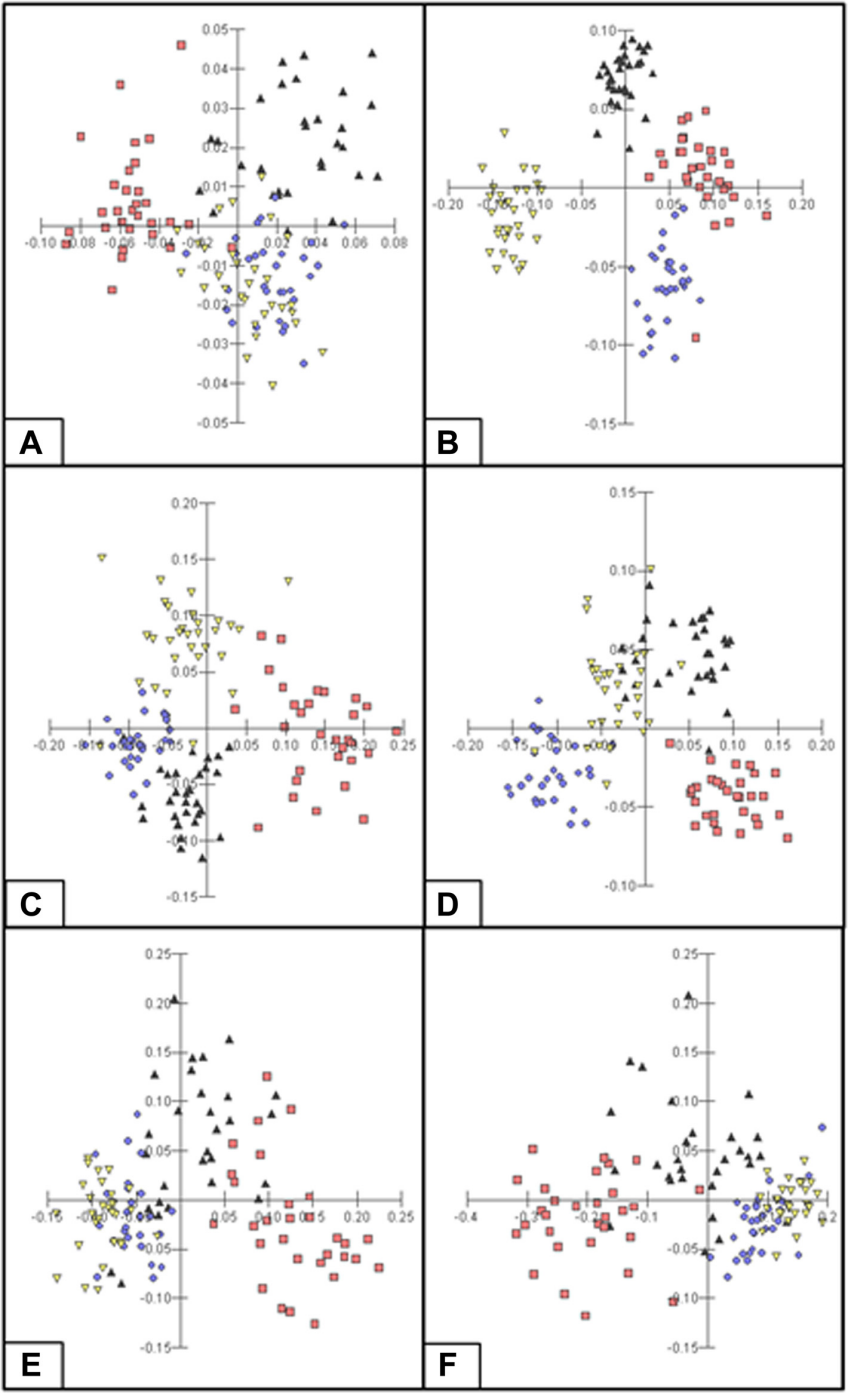
Discriminant analysis of shell morphometry of *Leptinaria unilamellata* from localities of Juiz de Fora (black triangle symbol), Cruzília (red square symbol), Barra Mansa (violet circle symbol) and Floriano (yellow triangle symbol), with ages of 30 **(A)**, 60 **(B)**, 90 **(C)**, 120 **(D)**, 150 **(E)** and 180 **(F)** days.

The discriminant analysis (Figure 4) showed at the age of 30 days the formation of three poorly defined groups. At the age of 60, 90 and 120 days four well defined groups, corresponding to each morphotype, were formed. Finally, at the ages of 150 and 180 days the results of the discriminant analysis were more similar to those found in the previous analyses of interpopulational variability.

### Growth allometry

The relationships 4: H = a . W^b^ (Shell height = a . Shell width^b^) and 5: SH = a . H^b^ (Spire height = a . Shell height^b^) provided more significant allometric coefficient values (Table 5).

**Table 5:** **Allometric relationships between linear measurements taken from individuals of** ***Leptinaria unilamellata*** **from Juiz de Fora, Cruzília, Barra Mansa and Floriano locations at the age of 180 days**

**Locations**	**Equations**	**Coefficient of determination**	**Standard error**	**Test-t**	**Allometric relationship**
Shell aperture width (AW) and shell width (W)					
JF	AW = −0.25 . W^1.12^	0.37	0.116	t = 4.03 p = 0.0000	allometry +
FLO	AW = −0.63 . W ^-0.19^	0.005	0.0649	t = −0.37 p = 0.7135	allometry -
CR	AW = −0.36 . W ^0.13^	0.0076	0.1022	t = 0.46 p = 0.6466	allometry -
BM	AW = 0.26 . W ^0.05^	0.0006	0.0519	t = 0.13 p = 0.8949	allometry -
Shell aperture height (AH) and shell width (W)					
JF	AH = −0.14 . W^0.73^	0.5026	0.0279	t = 5.32 p = 0.0000	allometry -
FLO	AH = −0.21 . W^0.56^	0.1708	0.013	t = 2.40 p = 0.0231	allometry -
CR	AH = −0.11 . W^0.74^	0.2735	0.0612	t = 3.25 p = 0.0030	allometry -
BM	AH = 0.28 . W^0.32^	0.0508	0.028	t = 1.22 p = 0.2312	allometry -
Spire height (SH) and shell width (W)					
JF	SH = −0.03 . W^1.10^	0.5936	0.0442	6.40 p = 0.0000	allometry +
FLO	SH = −0.09 . W^0.80^	0.1904	0.0229	2.57 p = 0.0159	allometry -
CR	SH = −0.18 . W^0.34^	0.1716	0.0229	2.41 p = 0.0228	allometry -
BM	SH = 0.18 . W^0.58^	0.0708	0.0665	1.46 p = 0.1553	allometry -
Shell height (H) and shell width (W)					
JF	H = 0.29 . W^0.82^	0.6763	0.0171	7.65 p = 0.0000	allometry -
FLO	H = 0.26 . W^0.69^	0.2909	0.0098	3.39 p = 0.0021	allometry -
CR	H = 0.23 . W^0.36^	0.3421	0.0102	3.82 p = 0.0007	allometry -
BM	H = 0.78 . W^0.79^	0.1037	0.0806	1.80 p = 0.0825	allometry -
Spire height (SH) and shell height (H)					
JF	SH = −0.39 . H^1.11^	0.6034	0.0432	6.53 p = 0.0000	allometry +
FLO	SH = −0.40 . H^1.27^	0.7935	0.0059	10.37 p = 0.0000	allometry +
CR	SH = −0.42 . H^1.05^	0.6195	0.0105	6.75 p = 0.0000	allometry +
BM	SH = −0.32 . H^0.68^	0.5775	0.0302	6.19 p = 0.0001	allometry -
Shell aperture height (AH) and shell height (H)					
JF	AH = −0.37 . H^0.65^	0.3886	0.0338	4.31 p = 0.0000	allometry -
FLO	AH = −0.41 . H^0.45^	0.1765	0.0129	2.45 p = 0.0208	allometry -
CR	AH = −0.47 . H^1.34^	0.3309	0.0564	3.72 p = 0.0009	allometry +
BM	AH = 0.44 . H^0.01^	0	0.0295	0.03 p = 0.9754	allometry -

Legend: JF - snails from Juiz de Fora; CR - snails from Cruzília; BM - snails from Barra Mansa; FLO - snails from Floriano.

In the relationship 1, AW = a . W^b^ (Shell aperture width = a . Shell width^b^), except for Juiz de Fora shells, all the others morphotypes, in most of the ages showed negative allometry, where the growth rate of AW was inferior to the growth rate of W. Only at the age groups of 90 and 150 days, the growth pattern of shell’s linear measurements analyzed by this relationship was similar among the locations. The shells from the all locations had high frequency of negative allometry for the relation 2, AH = a . W^b^ (Shell aperture height = a . Shell width^b^), which means that the growth rate of AH was inferior to the SW rate. As a result of the relationship 3, SH = a . W^b^ (Spire height = a . Shell width^b^), there was a greater number of negative allometric relationships than positive ones. For the relationship 4, H = a . W^b^ (Shell height = a . Shell width^b^), negative allometry occurred in all of the analyses, where the growth rate of H was inferior to the growth rate of W. For the relationship 5, SH = a . H^b^ (Spire height = a . Shell height^b^), most of the growth patterns were allometric positive. The spire growth rate was greater than the shell height growth rate (spire more body whorl). At the most of the results in the relationship 6, AH = a . H^b^ (Shell aperture height = a . Shell height^b^) corresponded to negative allometric growth. The aperture height presented an inferior growth rate compared to the shell height (Table 5).

## Discussion

In the present study it was possible to demonstrate the shell morphology variability between *L. unilamellata* from different populations kept under the same laboratorial conditions. Conde- Padín et al. (2009), studying two ecotypes of the sea gastropod *Littorina saxatilis* adapted to different coastal habitats, at the same location, observed that differences in shell aperture, globosity and dimension were maintained when the snails are kept under laboratory conditions and asserts that these differences are determined by inherited differences. In the present study, shell height, shell aperture height and body whorl height were significantly different and therefore, are the variables with more robustness for morphotypes distinction.

The morphotypes from Barra Mansa and Floriano showed greater similarity during their development compared to the other locations. It is probable that snails from these two have greater genetic similarity, due to the possibility of gene flow related to the small geographical distance between these locations (21.3 km). However, the greater values for Barra Mansa snails in some ratios resulted in a more slender shell shape and a less globular body whorl when compared to the Floriano shells. The greater number of whorls and the similarity only in the penultimate whorl in Juiz de Fora shells in relation to Barra Mansa and Floriano, lead to the conclusion that for the Juiz de Fora shells the whorls prior to the penultimate whorl were proportionally smaller and the decrease in the whorl height in the spire is more pronounced compared to Floriano and Barra Mansa. Also, the greater values of body whorl in spire height/body whorl height ratio Juiz de Fora shells indicated a more globular shell when compared with these two locations. The globular shape could be interpreted as an effect of the larger shell width found in the Juiz de Fora shells, which results in a shell height/shell width ratio significantly smaller compared to the ratios observed for Floriano and Barra Mansa. As a consequence, the body whorl is wide and globular, with a deep suture between the penultimate whorl and body whorl.

The measurement average values of Cruzília shells were closer to the values observed in Juiz de Fora morphotype. However, the ratio spire height/body whorl height was more similar to that found for Barra Mansa and Floriano shells and presented a smaller number of whorls compared to Juiz de Fora shells. Therefore, it can be concluded that the whorls heights prior to the penultimate whorl of Cruzília shells are proportionally larger, when compared to the Juiz de Fora shells and, therefore, present a more gradual decrease towards the protoconch. However, the Cruzília shells are not as slender as the Barra Mansa and Floriano shells because they present a significantly greater shell width, which reflects in the smaller values of the ratio shell height/shell width. This results in a more globular shell, with a wide body whorl and a deep suture between the penultimate whorl and body whorl.

The relationships presented here demonstrate that the globular shape of the Juiz de Fora and Cruzília shells and the slender shape of the Barra Mansa and Floriano shells are due to the body whorl dimensions, expressed as the ratio body whorl height/shell width, and the relative proportions of body whorl and spire, expressed as the ratios shell height/shell width and spire height/body whorl height. The globular shape of Juiz de Fora and Cruzília shells are due the combination of the larger body whorl dimensions, relative body whorl and spire proportions and a rounded aperture. The oval aperture of Barra Mansa and Floriano shells is associated with the slender shell shape and, therefore, the values of the ratio shell height/shell width are greater than those observed in Juiz de Fora and Cruzília shells.

The Euclidean distance analysis showed that the similarity pattern between morphotypes was established after they reached sexual maturity (approximately at 90 days according to Carvalho et al. 2009). The relationships between the linear measurements varied between the locations, at the ages of 30 and 60 days, reflecting the existence of differences in shell during the initial phase of snails’ post-embryonic ontogeny. In the present study, it was possible to observe that the Barra Mansa and Floriano morphotypes became more similar throughout the snails’ development . The smaller distance differences between these two localities in cluster analysis at 150 and 180 days confirmed that at the adult phase, the relationships between the variables have a tendency to stabilize. In the juvenile phase (30 days), the discrimant analysis showed poorly defined groups, this fact indicates that the morphometric patterns at this phase were not yet well established. Based on the results found, Barra Mansa and Floriano shells exhibited a strong association and Cruzília and Juiz de Fora shells demonstrated to be similar groups, but not so close as the other two groups.

Among the six allometric relationships analyzed, the relationships 4 and 5 were more relevant to the morphotypes differentiation. The relationships 1 (growth rate of AW was inferior to the growth rate of W) and 2 (growth rate of AH was inferior to the SW rate) indicate that throughout the snails’ development, the growth in the shell width is not proportional to the increase in width and length of the shell aperture. Therefore, the shell aperture of young snails is larger relative to its shell size, when compared to the adult that possess a smaller opening in relation to shell width. The result of relationship 2 also demonstrates that an increase in shell width is not accompanied by an increase in shell aperture.

As the shell grows, the new whorl produced constitutes the body whorl until the formation of the next whorl. The body whorl always increases more in width than in height and, therefore, the maximum shell width corresponds to the largest body whorl width. The greater growth rate in the maximum shell width in relation to the total shell height, as demonstrated by the relationship 4, can be explained by the asymmetrical growth of body whorl, which makes the contribution of body whorl to the increase of total shell height (body whorl more spire), less significant than its contribution for the growth in shell width. The patterns expressed by relationship 3, in which the spire growth rate is superior to the width growth rate and for relationship 4, where there is a progressive expansion in width as the shell increases resulting in a conical form with broad base, a common feature of *L. unilamellata* shell. The relationship 5 (spire growth rate was greater than the shell height growth rate) also confirmed that the contribution of body whorl to the increase in shell height is less significant than the contribution for the increase in width.

The relationship 6 (aperture height presented an inferior growth rate compared to the shell height) can be explained by the fact that the shell growth of *L. unilamellata* occurs by the addition of material at the edge of the shell aperture in a downward direction, as a result, the next whorl produced, only partially overlaps the previous whorl. Thus, the body whorl height is always greater than the shell aperture height. In addition to the shell height which corresponds to the aperture height added to the penultimate whorl height. This growth pattern also contributes to the conical shape of the shell.

Molecular evidences have demonstrated that in isolated populations of some terrestrial species breeding occurs predominantly by self-fertilization (Backeljau et al. 2001; Heller 2001). Moreover, these animals have very low dispersion capacity, leading to the decrease of gene flow among populations. These characteristics may favor reproductive isolation intensifying geographical differentiation among land snails populations (Backeljau et al. 2001; Heller 2001; Pfenninger and Posada 2002; Chiba 2005).

The snails from Barra Mansa and Floriano, locations with smaller precipitation index (1198 mm/year) (IBGE 2011), presented smaller shell aperture values when compared to the snails from Cruzília and Juiz de Fora with higher precipitation indexes of 1568 mm/year and 1536 mm/year (IBGE 2011), respectively.

Among the shell morphology describers, the spire index and shell aperture size are considered as adaptive characters (Cain 1977; Chiba and Davison 2007). The small shell aperture area has been interpreted as an adaptation to reduce water loss during aestivation (Santos and Monteirom 2001; Fiorentino et al. 2008). Concerning spire index, Okajima and Chiba (2009) observed that shells with low-spired are better balanced and more adapted to locomotion on horizontal surfaces. In the present study, *L. unilamellata* from all localities presented a spire index values greater than 1.4 (data not shown). This species lives associated to the litter and more frequently accomplishes horizontal locomotion. According to the proposition of Okajima and Chiba (2009), the spiral indexes observed for *L. unilamellata* are not appropriate for the horizontal displacement. However, since these snails comprise small dimensions, the gravity effect on the shell balance is not sufficient to disturb locomotion.

Pressures related to reproductive strategies can also lead to selection of shells with a more or less wide aperture and greater or smaller spire index. The dimension of aperture is directly related to the diameter of eggs and, therefore, a narrow aperture limits the possibility of reproduction by ovoviviparity associated with K-strategy. On the other hand, narrower apertures offer smaller desiccation risk (Suvorov 2002). Thus, there is potentially a trade-off between reproductive investment and resistance to desiccation.

In the present study, the allometry relationship between shell aperture width and shell width, as well as the relationship between the shell aperture height and shell width, indicate that, during the snails’ development, the increase in shell width is not proportional to the increase of the shell aperture width and height. Thus, there is possibly an antagonism between the adoption of K-strategy and protection against desiccation, since the increase of the protecting properties of shell lead to the narrowing of the aperture, as stated by Vermeij (2002).

The results lead to two important questions. The first one: are the two morphological patterns observed (ie: slender shells and globular shells) related to trade-offs between reproductive strategy and protection against desiccation? The morphological differences and the variation in patterns of shell ontogeny were sustained among the snails from different populations, kept under the same laboratorial conditions. This fact suggests that such characteristics are primarily under genetic determination, which leads to the second question: what are the relative contributions of developmental plasticity in *L. unilamellata* and genetic differentiation among populations through natural selection?

Assuming the existence of an antagonism between reproductive strategy and protection against desiccation and also that natural selection is more important than developmental plasticity, we can hypothesizes the following scenario. When the pressure related to desiccation risk is less intense, phenotypes characterized by wider body whorl (and consequently wider oviduct) and greater shell aperture area may be favored, since this morphological pattern permits the production of a greater number of offspring, possibly with greater dimensions. When the desiccation risk is more intense, the trade-off between reproductive investment and protection against desiccation is more significant, and this may favor snails with slender shells and narrow apertures.

Other neotropical subulinid species, present elongated shells, with narrow apertures and high spire indices. The life history of these species is characterized by indeterminate growth, ovoviviparity and the release of calcified eggs (Dundee 1986; D’ávila and Bessa 2005a, b). Although *L. unilamellata* is also an ovoviviparous species with indeterminate growth, this species releases juveniles instead of eggs and present a more voluminous oviduct, compared to the other species. The shell shape of *L. unilamellata* is also rather different, presenting a more wide body whorl, which makes the shell more globular. It is possible that the shell shape as well the morphology of the oviduct reflects the reproductive strategy of *L. unilamellata.*

Since the spire indices of *L. unilamella* morphotypes cannot be explained by physical functional aspects (ie.: shell balance), the most likely explanation is the reproductive strategy of this species. Carvalho et al*.* (2009) found positive relationships between the number of embryos and shell size; number of embryos and shell whorls number; number of embryos and palial oviduct size; shell size and oviduct size; albumen gland size and shell size. Therefore, for *L. unilamellata*, having a high spired globose shell associated with indeterminate growth is a characteristic very important in terms of reproductive success. These results, associated to the fact that the pattern of similarity between the snails was established after they reached sexual maturity, may be an evidence that reproductive strategy is an important factor determining shell shape in *L. unilamellata*.

The shell aperture of the juvenile *L. unilamellata* is larger in relation to its corporal size, when compared to the adult, increasing the desiccation risk. The largest mortality in the juvenile phase is commonly verified in terrestrial snails (Almeida and Bessa 2001; Carvalho et al. 2008). However, the *L. unilamellata* reproductive strategy, with development of nestlings through ovoviviparity, including the possibility of nestlings’ retention in the oviduct of the parental snail, in response to adverse environmental conditions can favor the survival of the nestlings (Carvalho et al. 2009).

The results of allometric analysis allowed the discussion of some interesting points. In a few ages there were similarities in growth allometry between the morphotypes, indicating that not only the final adult shell shape is different among the morphotypes, but also the whole process of shell formation.

For the snails with indeterminate growth, species distinction is difficult during the juvenile phase, due to the great similarity of shell morphometric patterns. Since a reflected outer lip is not formed when the snail reaches maturity, the juvenile body whorl possesses the same shape of an adult individual (Raup 1961). Dutra (1988) observed that shell characteristics of *L. unilamellata*, such as a simple outer lip, parietal lamella and truncate columella are already present in the embryo. Carvalho et al*.* (2009) confirmed that, juvenile individuals of this species possess shells which are similar to those of adult individuals in the shape of aperture and parietal lamella. It is possible that shell characteristics, considered important for taxonomy can also be present from the initial developmental phases in other species of genus *Leptinaria*. In this sense, an in-depth revision of this genus is necessary, while taking into account other morphologic characteristics.

## References

[CR1] Almeida MN, Bessa ECA (2001). Estudo do crescimento e da reprodução de *Leptinaria unilamellata* (D’orbigny) (Mollusca, Subulinidae) em laboratório. Rev Bras Zool.

[CR2] Araújo JLB (1982). Alguns moluscos terrestres como hospedeiros intermediários de parasitos de animais domésticos, no Brasil: estudos sobre a anatomia, sistemática e participação em helmintoses.

[CR3] Backeljau T, Baur A, Baur B, Barker GM (2001). Population and Conservation Genetics. The Biology of Terrestrial Molluscs.

[CR4] Bessa ECA, Araújo JLB (1995). Oviposição, tamanho de ovos e medida do comprimento da concha em diferentes fases do desenvolvimento de *Subulina octona* (Brugüière) (Pulmonata, Subulinidae) em condições de laboratório. Rev Bras Zool.

[CR5] Bessa ECA, Araújo JLB (1995). Ocorrência de autofecundação em *Subulina octona* (Brugüière) (Pulmonata, Subulinidae) em condições de laboratório. Rev Bras Zool.

[CR6] Brandolini SVPB, Gomes APS (2002). Influência de diferentes dietas sobre o crescimento, sobrevivência e reprodução de *Leptinaria unilamellata* (d’Orbigny, 1835) (Gastropoda, Subulinidae) em laboratório. Rev Bras Zoociênc.

[CR7] Cain AJ (1977). Variation in spire index of some coiled gastropod shells, and its evolutionary significance. Philos T Roy Soc B.

[CR8] Carstensen D, Laudien J, Leese F, Arntz W, Held C (2009). Genetic variability, shell and sperm morphology suggest that the surf clams *Donax marincovichi* and *D. obesulus* are one species. J Mollus Studies.

[CR9] Carvajal-Rodriguez A, Conde-Padín P, Rolan-Alvarez E (2005). Decomposing shell form into size and shape by geometric morphometric methods in two sympatric ecotypes of *Littorina saxatilis*. J Mollus Studies.

[CR10] Carvalho CM, Bessa ECA, D’ávila S (2008). Life history strategy of *Bradybaena similaris* (Ferussac, 1821) (Mollusca, Pulmonata, Bradybaenidae). Molluscan Res.

[CR11] Carvalho CM, Silva JP, Mendonça CLF, Bessa ECA, D’ávila S (2009). Life history strategy of *Leptinaria unilamellata* (d’Orbigny, 1835) (Mollusca, Pulmonata, Subulinidae). Inver Rep Dev.

[CR12] Chiba S (2005). Appearance of morphological novelty in a hybrid zone between two species of land snail. Evolution.

[CR13] Chiba S, Davison A (2007). Shell shape and habitat use in the North-west Pacific land snail *Mandarina polita* from Hahajima, Ogasawara: current adaptation or ghost of species past?. Biol J Linn Soc.

[CR14] Chiu YW, Hon-Cheng C, Sin-Che L, Chen CA (2002). Morphometric analysis of shell and operculum variations in the viviparid snail, *Cipangopaludina chinensis* (Mollusca: Gastropoda). Zool Stud.

[CR15] Choh MS, Yap CK, Tan SG, Jambari HA (2006). Morphological and allozyme studies of small terrestrial snails (*Opeas* sp., *Subulina* sp., and *Huttonella bicolor*) collected from Peninsular Malaysia. Russ J Genet.

[CR16] Conde- Padín P, Grahame JW, Rolán-Alvarez E (2007). Detecting shape differences in species of the *Littorina saxatilis* complex by morphometric analysis. J Mollus Studies.

[CR17] Conde- Padín P, Caballero A, Rolán-Alvarez E (2009). Relative role of genetic determination and plastic response during ontogeny for shell-shape traits subjected to diversifying selection. Evolution.

[CR18] D’ávila S, Bessa ECA (2005). Influência do substrato sobre a reprodução de *Subulina octona* (Bruguière) (Mollusca, Subulinidae), sob condições de laboratório. Rev Bras Zool.

[CR19] D’ávila S, Bessa ECA (2005). Influência do substrato sobre o crescimento de *Subulina octona* (Bruguière) (Mollusca, Subulinidae), sob condições de laboratório. Rev Bras Zool.

[CR20] Dewitt TJ, Sih A, Hucko A (1999). Trait compensation and cospecialization in a freshwater snail: size, shape and antipredator behaviour. Anim Behav.

[CR21] Dundee DS (1986). Notes on the habits and anatomy of the introduced land snails, *Rumina* and *Lamellaxis* (Subulinidae). Nautilus.

[CR22] Dutra AVC (1988). Aspectos da ecologia e da reprodução de *Leptinaria unilamellata* (Orbigny, 1835) (Gastropoda, Subulinidae). Revista Brasileira de Zoologia.

[CR23] Fiorentino V, Manganelli G, Giusti F (2008). Multiple scale patterns of shell and anatomy variability in land snails: the case of the Sicilian *Marmorana* (Gastropoda: Pulmonata, Helicidae). Biol J Linn Soc.

[CR24] Heller J, Barker GM (2001). Life History Strategies. The Biology of Terrestrial Molluscs.

[CR25] Hórsak M, Hájek M, Díte D, Tichý L (2007). Modern distribution patterns of snails and plants in the western carpathian spring fens: is it a result of historical development?. J Mollus Studies.

[CR26] Instituto Brasileiro de Geografia e Estatística (IBGE) (2011) Divisão Territorial do Brasil e Limites Territoriais. Page visited in May 03, 2011. http://www.ibge.gov.br/home/.

[CR27] Medeiros C, Daniel PA, Santos EO, Ferreira PB, Caldeira RL, Mendonça CLF, Carvalho OS, D’ávila S (2013). Macro- and microscopic morphology of the reproductive system of *Leptinaria unilamellata* (d’Orbigny, 1835) (Mollusca: Pulmonata: Subulinidae). J Nat Hist.

[CR28] Okajima R, Chiba S (2009). Cause of bimodal distribution in the shape of a terrestrial gastropod. Evolution.

[CR29] Paraense WL (1976). *Lymnaea viatrix*: a study of topotypic specimens (Mollusca: Lymnaeidae). Rev Bras Biol.

[CR30] Patel NH (2009). Asimetry with a twist. Nature.

[CR31] Pfenninger M, Posada D (2002). Phylogeographic history of the land snail *Candidula unifasciata* (Helicellinae, Stylommatophora): Fragmentation, corridor migration, and secondary contact. Evolution.

[CR32] Raahauge P, Kristensen TK (2000). A comparison of *Bulinus africanus* group species (Planorbidae; Gastropoda) by use of the internal transcribed spacer 1 region combined by morphological and anatomical characters. Acta Trop.

[CR33] Raup DM (1961). Geometry of coiling in gastropods. P Natl Acad Sci Usa.

[CR34] Santos SB, Monteirom DP (2001). Composição de gastrópodes terrestres em duas áreas do Centro de Estudos Ambientais e Desenvolvimento Sustentado (CEADS), Vila Dois Rios, Ilha Grande, Rio de Janeiro, Brasil – um estudo-piloto. Rev Bras Zool.

[CR35] Simone LRL (2006). Land and Freshwater Molluscs of Brazil.

[CR36] Suvorov AN (2002). Prospects for studies of morphological variability of land pulmonate snails. Biol Bull.

[CR37] Tryon GW, Pilsbry HA (1906) Manual of Conchology. Conchology Dept. Academy Natural Sciences, Philadelphia. 18(3):289.

[CR38] Velasquez LE (2006). Synonymy between *Lymnaea bogotensis* Pilsbry, 1935 and *Lymnaea cousini* Jousseaume, 1887 (Gastropoda: Lymnaeidae). Mem Inst Oswaldo Cruz.

[CR39] Vermeij GJ (2002). Characters in context: molluscan shells and the forces that mold them. Paleobiology.

[CR40] Wada S, Chiba S (2013). The dual protection of a micro land snail against a micro predatory snail. PLoS ONE.

[CR41] Wilke T, Pfenninger M, Davis GM (2002). Anatomical variation in cryptic mudsnail species: statistical discrimination and evolutionary significance. P Acad Nat Sci Phila.

